# Trends in Prescription Medications Reflect the Current Substance Misuse Trends

**DOI:** 10.3390/healthcare14213744

**Published:** 2026-11-03

**Authors:** Einat Peles, Miriam Adelson, Shaul Schreiber

**Affiliations:** 1 Dr. Miriam & Sheldon G. Adelson Clinic for Drug Abuse, Treatment & Research, Division of Psychiatry, Tel Aviv Sourasky University Medical Center, Tel Aviv 6423906, Israel; miriam.adelson@sands.com (M.A.); shaulsch@tlvmc.gov.il (S.S.); 2 Gray Faculty of Medical and Health Sciences, Tel Aviv University, Tel Aviv 6997801, Israel; 3 Sagol School of Neuroscience, Tel Aviv University, Tel Aviv 6997801, Israel

**Keywords:** fentanyl, pregabalin, methadone maintenance, trends, misuse

## Abstract

**Background**: In Israel, fentanyl and pregabalin prescription rates for pain management have increased over the years. However, Health Maintenance Organizations (HMOs) implemented restrictions on opioid prescribing in 2022, potentially reducing fentanyl availability while pregabalin prescribing remained unrestricted. We aimed to examine temporal trends in fentanyl and pregabalin misuse between 2021 and 2025 among individuals receiving methadone maintenance treatment (MMT) in Tel Aviv, in the context of changes in opioid prescribing policies, and to characterize fentanyl misuse groups. **Methods**: In this prospective descriptive study, between February 2021 and December 2025, patients (ranging from 291 to 311) were screened three to four times annually for fentanyl and pregabalin, in addition to other routine urine tests for substance use. Trends in positive urine screens for pregabalin and fentanyl were compared. Additionally, methadone doses and serum methadone levels were compared among patients who consistently tested positive for fentanyl (*n* = 15), tested negative for fentanyl (*n* = 202), or Stopped/Started fentanyl Use (*n* = 22) between 2023 and 2025. **Results**: Pregabalin positivity ranged between 11.2% and 15.9%, with no temporal trend. The fentanyl positivity rate increased from 9.8% in October 2021 to 15.1% in March 2023 and subsequently decreased to 7.5% in March 2025, paralleling the reported reduction in fentanyl availability following HMO restrictions. The methadone dose among patients testing positive for fentanyl was higher, whether they discontinued or continued fentanyl use, than among patients who consistently tested negative for fentanyl. **Conclusions**: Fentanyl positivity decreased following HMO restrictions and the associated reduction in fentanyl availability, whereas pregabalin positivity remained stable during the study period. Further research is needed to identify the distinct drivers of pregabalin misuse and evaluate appropriate prevention and prescribing strategies. Patients with fentanyl-positive urine screens may require higher methadone doses.

## 1. Introduction

The opioid epidemic in the United States and other countries, where the prescribing of opioids for chronic, non-cancer pain was vigorously advocated, thereby facilitating pervasive diversion and misuse, has been well documented [1]. Pain is defined as an unpleasant sensory and emotional experience associated with actual or potential tissue damage or resembling that associated with such damage [2]. During the 1990s, efforts by the American Pain Society, the International Association for the Study of Pain, and the Joint Commission on Accreditation of Healthcare Organizations to promote pain as the “fifth vital sign” unintentionally encouraged inappropriate opioid prescribing, contributing to the opioid epidemic [3,4,5].

The opioid epidemic was also reflected in Israel in an increase in opioid prescribing; between 2012 and 2015, opioid consumption increased by 125%, the largest rise among the Organization for Economic Co-operation and Development countries [6], followed by a dramatic increase in fentanyl prescriptions [7]. Data from Israel’s largest healthcare provider, Clalit Health Services, showed a further increase in outpatient opioid consumption between 2016 and 2018, largely driven by fentanyl use among non-elderly patients without malignancy [7]. However, unlike in the USA, there is no evidence of a large-scale illicit or diverted fentanyl market in Israel. Fentanyl is typically prescribed by physicians as a potent pain reliever for serious medical conditions, and non-medical use often arises from diversion, misuse of prescriptions, or sharing of legally obtained patches and other formulations.

As with fentanyl, and consistent with reports from other countries [8], prescription rates of the non-opioid gabapentinoid analgesic pregabalin in Israel increased substantially between 2009 and 2019 [9].

MMT is the most effective treatment for opioid use disorder (OUD) [10,11]. As OUD is a chronic, relapsing disorder, the use of opioids and other substances is routinely screened as part of treatment. According to the Israeli Ministry of Health’s 2004 guidelines, the use of opioids, including morphine, heroin, and codeine, as well as cocaine, benzodiazepines, amphetamines, and cannabis, is routinely monitored in MMT. Patients receive an individually tailored adequate methadone dose, defined as the minimum individualized dose that successfully prevents withdrawal symptoms and overdose, blocks mu-opioid receptors, reduces craving, stabilizes the hypothalamic–pituitary–adrenal axis, and facilitates opioid abstinence, as reflected by negative urine opioid tests [10,12]. In addition, monitoring non-opioid substance use is also important, primarily to assess patients’ rehabilitation progress and to inform decisions regarding treatment privileges. Patients may be granted take-home methadone doses (THDs) based on complete and sustained abstinence from substance use, as indicated by negative urine screening results. Therefore, monitoring protocols are periodically updated to include newly emerging substances, in addition to routinely screened substances.

Due to the substantial increase in fentanyl [7,13] and pregabalin [9] prescriptions in Israel, the MMT clinic of a large tertiary university medical center in Tel Aviv implemented urine screening for these substances, as they were not included in routine urine screening. Specifically, all MMT patients were screened every quarter while those with THD privileges were tested monthly. Using data from this clinic, we previously reported an increase in the proportion of patients with fentanyl-positive urine tests from 9.8% in February 2021 to 15.1% in March 2023 [14], as well as a high prevalence of pregabalin misuse, with 17.7% of patients testing positive in December 2017 [15].

In July 2022, following guidelines issued by the Israeli Ministry of Health, HMOs were required to establish monitoring mechanisms for long-term opioid prescriptions. Specifically, HMOs were instructed to ensure that every patient receiving opioids for at least 90 days was referred for evaluation by a pain specialist. This policy change was soon followed by a significant reduction in the volume of opioid prescriptions [16].

We hypothesized that a reduction in fentanyl availability would be reflected in a decreased prevalence of fentanyl misuse among patients receiving MMT, whereas pregabalin misuse would remain relatively stable, given the absence of comparable regulatory changes affecting its prescribing.

Therefore, we specifically studied the following among individuals with OUD receiving MMT: (1) We investigated the prevalence and temporal trends of fentanyl and pregabalin misuse among all current patients receiving MMT between 2021 and 2025, along with their corresponding methadone doses. (2) In a follow-up analysis of patients who remained in treatment between 2023 and 2025, we compared the characteristics of patients who consistently tested positive for fentanyl, those who never tested positive, and those with discordant results, either starting or discontinuing fentanyl use.

## 2. Materials and Methods

### 2.1. Study Design

In a prospective descriptive analysis, we studied temporal trends in fentanyl and pregabalin misuse among all current patients receiving MMT between 2021 and 2025 (with 291–311 individuals assessed at each time point), as well as their corresponding methadone doses. For additional analyses, we studied 239 patients—all available patients who remained in MMT between 2023 and 2025 and had fentanyl and pregabalin evaluations in both March 2023 and March 2025. Of these, 228 had serum methadone levels measured in 2023 (their serum levels and methadone doses on the dates of serum evaluation are presented in Table 1), and 170 had additional serum levels measured in 2024 or 2025 (changes in their serum methadone levels and methadone doses between the two evaluations are presented in Table 2).

### 2.2. Study Population

The MMT clinic is accredited by the Commission on Accreditation of Rehabilitation Facilities and is part of a large, tertiary university medical center in Tel Aviv, Israel. The clinic was established in June 1993 and admits individuals aged ≥18 years who are diagnosed with OUD according to the Diagnostic and Statistical Manual (DSM-5 or DSM-IV-TR) and have undergone at least two unsuccessful inpatient detoxification attempts. Exclusion criteria were age <18 years, failure to meet the DSM diagnostic criteria for OUD, or fewer than two failed inpatient detoxification attempts. Since June 1993, 1039 new patients had initiated treatment by February 2021, when the study began, and 1196 by December 2025, when the study ended, with approximately 300 current patients each month.

Patients receive their methadone dose daily at the clinic and attend regular sessions with a designated personal therapist. Patients may earn the privilege of THD, up to a maximum of two weeks of THD. Specifically, the first THD can be achieved if urine screens are negative for all substances for at least 3 months, followed by additional monthly THD, up to one week, while two weeks of THD can be achieved following an additional two years of full abstinence (for details, see [17]).

### 2.3. Urine Toxicology

Patients in our MMT clinic undergo repeated observed, random urine testing, approximately twice monthly, throughout treatment for opioids, including morphine, heroin, codeine, the cocaine metabolite benzoylecgonine, benzodiazepine, cannabis, and the methadone metabolite, using enzyme immunoassay systems (DRI^®^ and CEDIA^®^) [18]. For each substance, a positive monthly result was defined as at least one positive urine sample during that month.

Since February 2021, in addition to the routine urine tests described above, additional substances—namely, fentanyl, oxycodone, tramadol, pregabalin, methylphenidate, and alcohol—were screened monthly among patients with THD using Rapid Test (JusCheck^®^) [19]. However, these additional substances were also screened in all current patients every 3–4 months, with three screening time points in 2021 (February, June, and October) and four screening time points annually from 2022 to 2025 (March, June, September, and December) for a total of 19 time points.

### 2.4. The Prevalence and Temporal Trends

Temporal trends were assessed using the 19 time points that were available for all substances among all current patients between 2021 and 2025, ranging from 291 to 311 individuals per time point. We further analyzed methadone doses at the beginning of each of the 19 time points.

### 2.5. Follow-Up Study Group

Patients who were consistently in MMT with available fentanyl urine screening results (*N* = 239) were analyzed for changes in methadone dose and serum levels. The patients were categorized according to their fentanyl urine-screening results in March 2023 and March 2025 into 3 groups: “Always”, “Never”, and “Started/Stopped”.

### 2.6. Methadone Dose and Serum Level

Methadone doses in March 2023 and March 2025, and the closest available serum methadone level to each time point, were used for analysis. Patients are routinely evaluated for serum methadone levels before receiving their first THD privilege. THD privileges were provided when patients were clinically stabilized on methadone, maintained on a stable methadone dose, and had no detectable opioids, cocaine, cannabis, or benzodiazepines in urine screening for at least three months. For patients receiving ≥150 mg/day, serum methadone levels are measured annually; for those receiving <150 mg/day, levels are measured biennially. Methadone concentrations were determined using gas chromatography–mass spectrometry (GC-MS; Clinical Science Laboratory, Mansfield, MA, USA).

### 2.7. Statistical Analysis

Statistical analyses were performed using IBM SPSS Statistics version 29. To evaluate temporal trends in the proportion of fentanyl, pregabalin, and other substances at 19 time points between 2021 and 2025, we used a repeated-measures generalized estimating equation (GEE) to assess binary and continuous outcomes, with patient ID specified as the subject variable and the time point specified as the within-subject variable. A binomial distribution with a logit link function was used to account for the binary urine test results and the correlation among repeated measurements from the same patient. Additionally, we used GEE for the methadone dose trend, with a continuous outcome assuming a normal distribution, while accounting for the binary pregabalin and fentanyl urine results.

Comparisons among fentanyl groups in March 2023 and March 2025, “Always”, “Never”, and “Started/Stopped”, were conducted using the chi-square or Fisher’s exact test for categorical variables and one-way analysis of variance (ANOVA) for continuous variables. When the assumption of homogeneity of variances was violated, Welch’s ANOVA was used. Bonferroni-adjusted post hoc comparisons were conducted following standard ANOVA, whereas Games–Howell post hoc comparisons were used following Welch’s ANOVA. Repeated-measures analyses were used to evaluate changes in methadone doses and serum methadone levels between March 2023 and March 2025, stratified by fentanyl groups.

## 3. Results

### 3.1. The Prevalence and Temporal Trends

#### Patient Characteristics

At study initiation, of the 291 current patients (February 2021), 21.6% were females, 52.6% had a history of opioid use for ≥ 20 years before admission to MMT, 26.1% had ≥ 12 years of education, 57% had ever injected drugs, 54.3% had antibodies to hepatitis C, and 6.2% had antibodies to HIV. At admission to MMT, in addition to opioids, 51.9% tested positive for benzodiazepines, 12.7% for cannabis, and 29% for cocaine.

### 3.2. Trends 2021 and 2025

**Figure 1:**
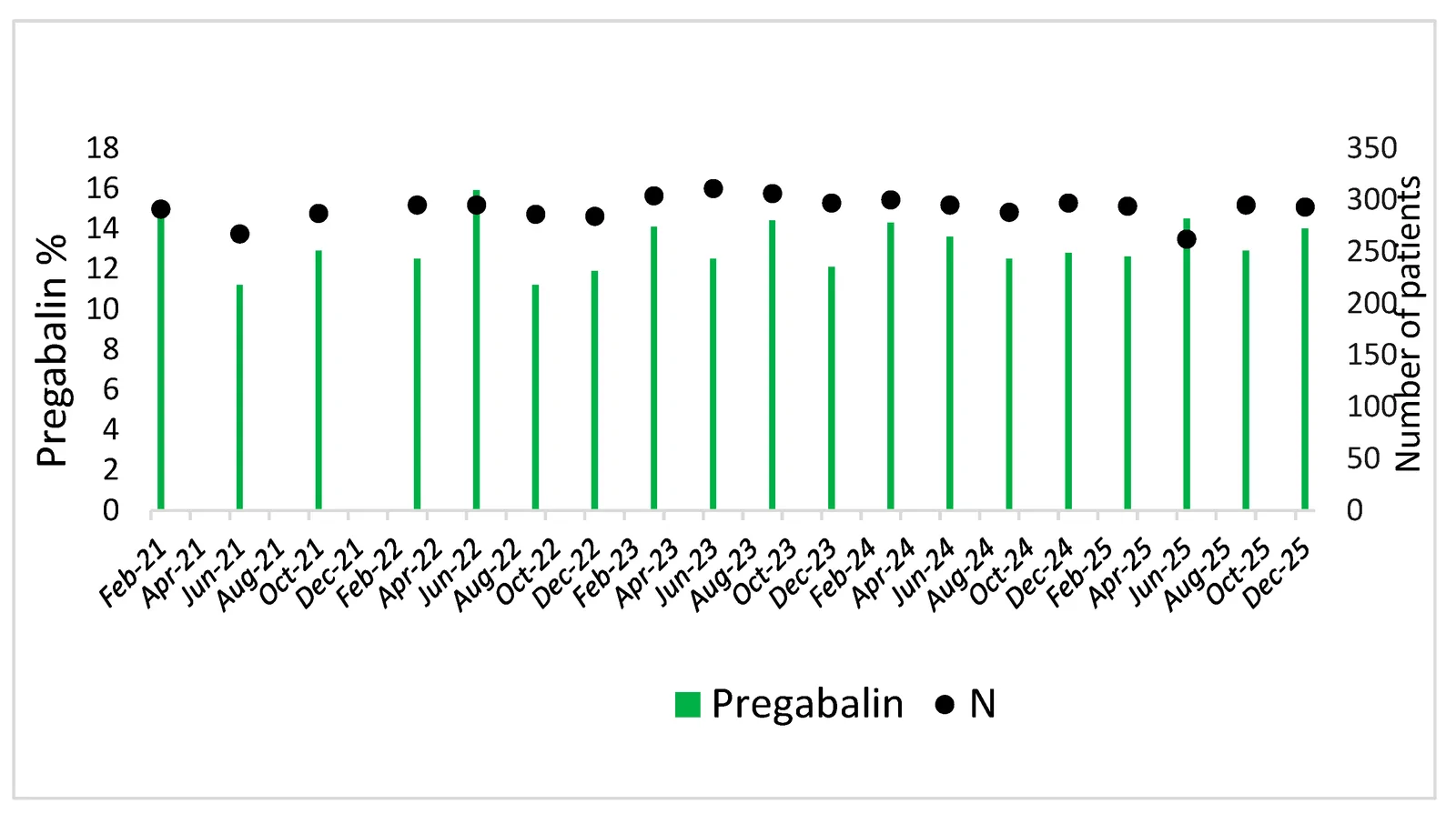
Pregabalin prevalence (%) among current MMT patients between 2021 and 2025, measured at each assessment time point. The number of patients assessed at each time point is reported.

**Figure 2:**
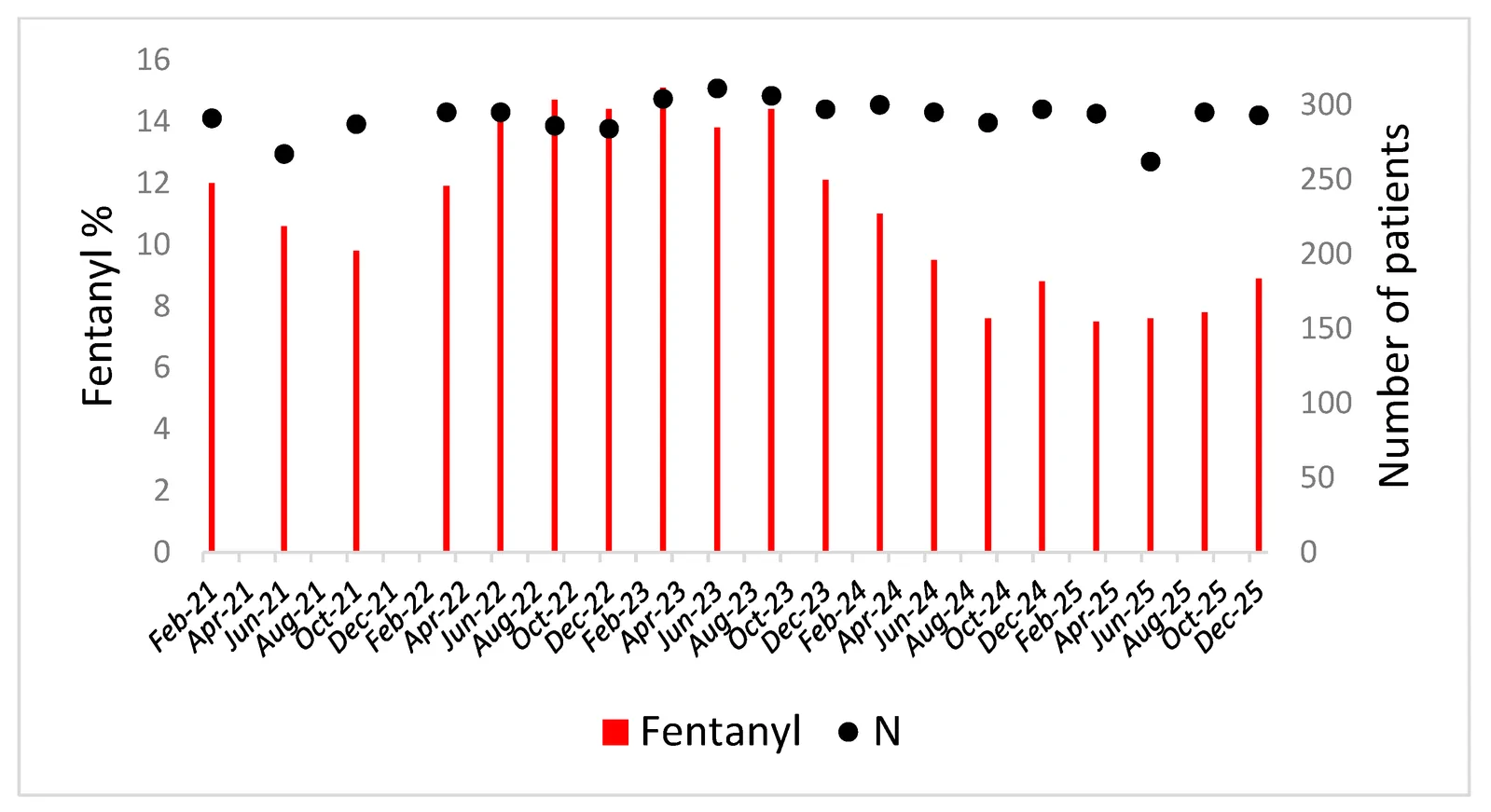
Fentanyl prevalence (%) among current MMT patients between 2021 and 2025, measured at each assessment time point. The number of patients assessed at each time point is reported.

The proportion of pregabalin and fentanyl between February 2021 and December 2025 is presented in Figure 1 and Figure 2.

Pregabalin positivity ranged between 11.2% and 15.9% with no significant temporal effect across 2021–2025 (Wald χ^2^ = 0.9, *p* = 0.9) (Figure 1). In contrast, a significant temporal effect on the proportion of fentanyl-positive urine screens was observed between 2021 and 2025 (Wald χ^2^ = 28.4, *p* < 0.001). Specifically, fentanyl positivity increased from a minimum of 9.8% in 2021 to a peak of 15.1% in 2023, followed by a significant decrease to 7.5% in 2025. The increase during 2021–2023 was significant (Wald χ^2^ = 13.7, *p* < 0.001), as was the subsequent decrease during 2023–2025 (Wald χ^2^ = 20.1, *p* < 0.001) (Figure 2).

The proportions of other routinely screened opioids at these time points ranged between 8.8% and 15.1% with no significant temporal effect across 2021–2025 (Wald χ^2^ = 4.9, *p* = 0.3). Similarly, no significant temporal effect was observed for oxycodone, which ranged between 0.3% and 2.4% (Wald χ^2^ = 5.2, *p* = 0.3). Tramadol positivity decreased significantly from 3.8% in February 2021 to 0% in June 2023. No significant trends were observed for methylphenidate, which ranged between 7.5% and 11.2%, Wald χ^2^ = 3.7, *p* = 0.4; cocaine, which ranged between 16.5% and 23.3%, Wald χ^2^ = 7.4, *p* = 0.1; or cannabis, which ranged between 10.9% and 15.1%, Wald χ^2^ = 3.6, *p* = 0.5. With respect to benzodiazepine, which ranged between 20.8 and 29.5%, a significant overall difference across years was observed when year was modeled as a categorical factor (Wald χ^2^ = 9.8, *p* = 0.04); however, no significant linear temporal trend was observed (B = 0.036, Wald χ^2^ = 1.3, *p* = 0.25).

**Figure 3:**
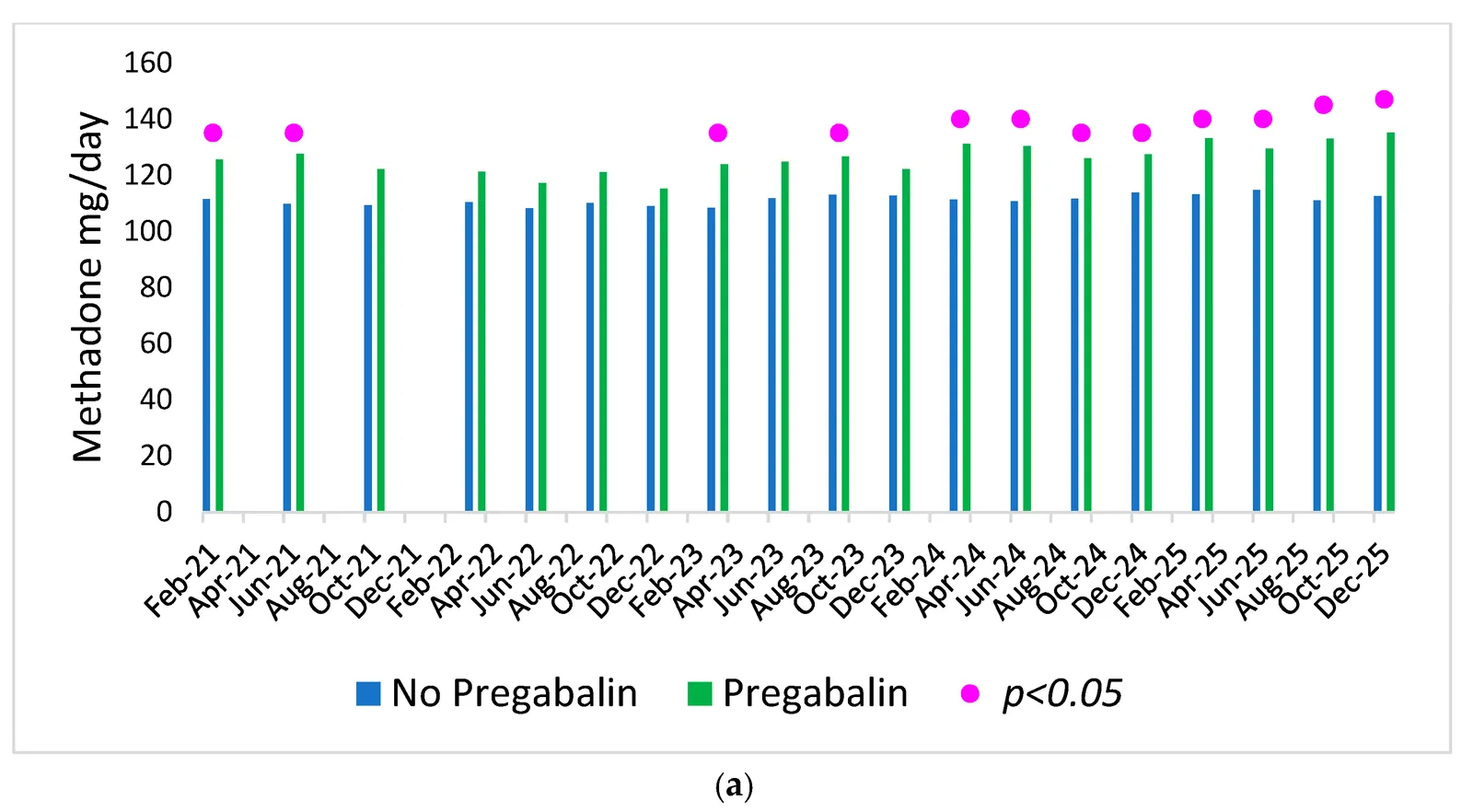

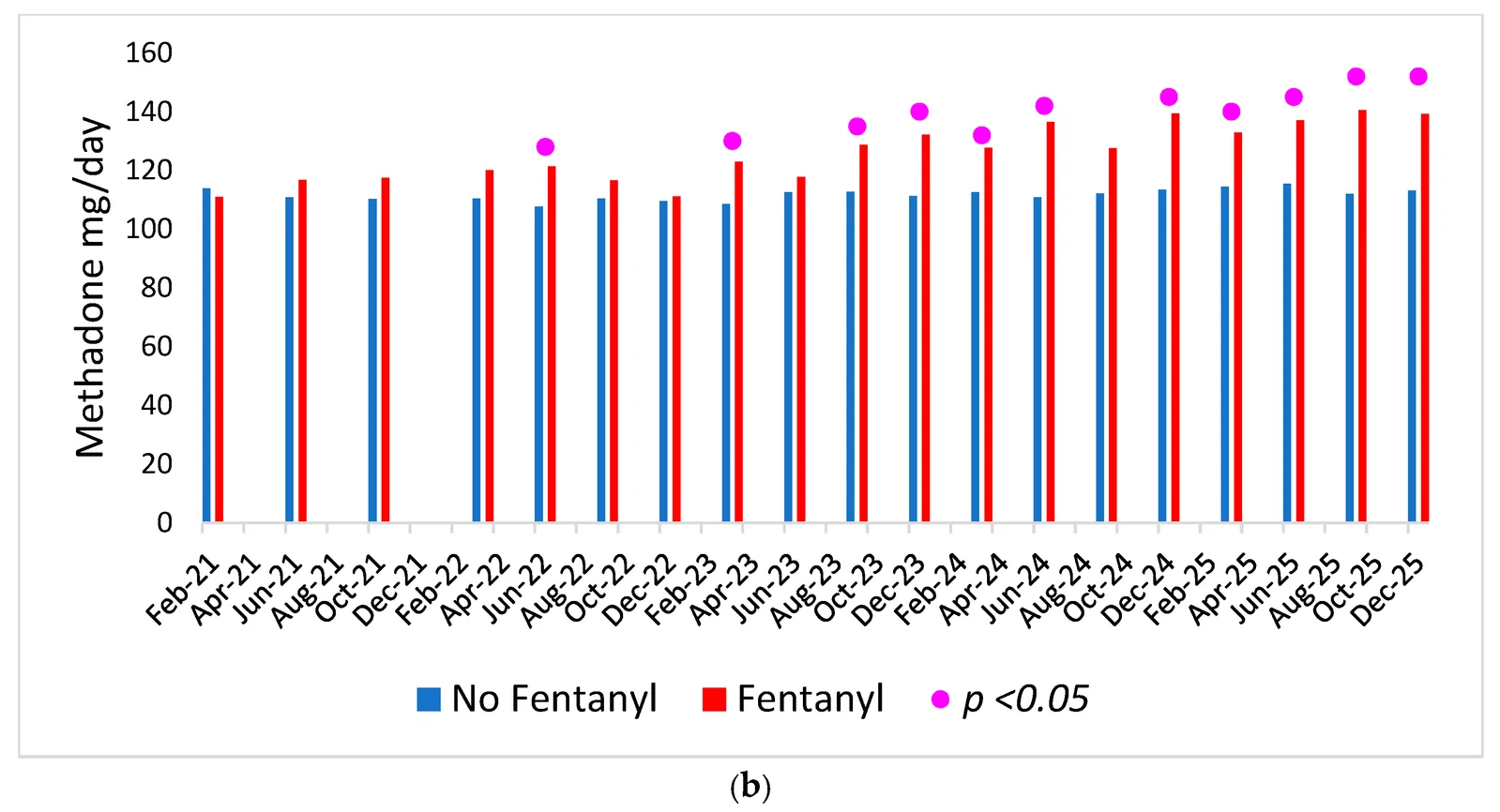
(**a**) Mean methadone dose from 2021 to 2025 at each assessment stratified by current pregabalin use. Significant differences (*p* < 0.05) in methadone dose between patients who tested positive and those who tested negative for pregabalin at individual assessment time points are indicated. (**b**) Mean methadone dose from 2021 to 2025 at each assessment stratified by current fentanyl use. Significant differences (*p* < 0.05) in methadone dose between patients who tested positive and those who tested negative for fentanyl at individual assessment time points are indicated.

Methadone dose over the study period, stratified by fentanyl and pregabalin urine-test status, is presented in Figure 3a,b.

Between 2021 and 2025, a significant temporal effect was observed for methadone dose (Wald χ^2^ = 9.5, *p* = 0.05), with a significant temporal increase when year was modeled as a continuous covariate (B = 1.2 mg/year, Wald χ^2^ = 4.7, *p* = 0.03). Including fentanyl in the GEE model, methadone dose increased significantly over time among fentanyl-positive patients (B = 6.26 mg/year, *p* < 0.001), whereas the increase was substantially smaller among fentanyl-negative patients (B = 0.75 mg/year). The difference in the slopes between the two groups was statistically significant (year × fentanyl interaction: B = −5.51, *p* = 0.001). In 2021, the methadone dose did not differ significantly between fentanyl-positive and fentanyl-negative patients (*p* = 0.602).

In a GEE model including pregabalin, patients with a pregabalin-positive urine test had significantly higher methadone doses (B = 2.89 mg, *p* = 0.025), but the temporal change in methadone dose did not differ significantly according to pregabalin status (year × pregabalin interaction: B = −2.09, *p* = 0.137).

In a GEE model including both fentanyl and pregabalin, the interaction between fentanyl and year remained significant (B = −5.54 mg/year, *p* < 0.001), with no significant temporal interaction for pregabalin (B = −1.39 mg/year, *p* = 0.31).

### 3.3. Follow-Up Study Group

#### 3.3.1. Patient Characteristics (Table 1)

The fentanyl group includes 15 patients who “Always” tested positive for fentanyl (March 2023 and March 2025), 202 who “Never” tested positive, and 22 patients with discordant results (18 discontinued fentanyl and tested positive only in March 2023, whereas 4 initiated fentanyl use and tested positive only in March 2025). The groups differed only by concomitant substance use. Specifically, the highest prevalence of concomitant substance use was observed among patients who consistently tested positive for fentanyl (benzodiazepine 46.7%, cannabis 33.3%, cocaine 40%, pregabalin 33.3%, methylphenidate 13.3%), followed by those who changed fentanyl status, predominantly those who discontinued fentanyl use, and the lowest prevalence among patients who never tested positive (benzodiazepine 15.8%, cannabis 7.9%, cocaine 17.8%, pregabalin 7.4%, methylphenidate 2.5%). Among those who had serum methadone levels measured (14 Always, 22 Stopped/Started, and 192 Never), methadone doses at the time of serum measurement differed significantly among the three fentanyl groups (Welch’s ANOVA, F(2, 27.9) = 7.3, *p* = 0.003). Games–Howell post hoc comparisons indicated that methadone doses were significantly lower in the “Never” group than in the “Always” group (adjusted *p* = 0.01), with a borderline difference between the “Never” and Stopped/Started groups (adjusted *p* = 0.05). Similarly, serum methadone levels were numerically lower in the Never group than in the Stopped/Started group; however, this difference was not statistically significant in the Games–Howell pairwise comparison (adjusted *p* = 0.171).

**Table 1:** Comparison between patient groups according to their fentanyl urine-screening results in March 2023 and March 2025.

	Positive “Always” *N* = 15 (%)	Stopped/Started *N* = 22 (%)	Negative “Never” *N* = 202 (%)	*p* Value
Female gender, %	1 (6.7)	5 (22.7)	50 (24.8)	0.2
Current age, y	56.2 ± 10.9	57.4 ± 7.7	56.5 ± 9.7	0.9
Age at admission, y	41.7 ± 11.7	44.0 ± 10.1	43.3 ± 11.5	0.8
Duration in MMT, y	14.3 ± 9.2	13.1 ± 8.3	12.8 ± 9.2	0.8
Opioid ≥20 y, %	10 (66.7)	11 (50.0)	101 (50.0)	0.5
Education ≥12 y, %	6 (40.0)	4 (18.2)	52 (25.7)	0.3
Drug injected, %	6 (40.0)	11 (50.0)	93 (46.0)	0.8
Hepatitis C Antibody, %	5 (33.3)	13 (59.1)	102 (50.5)	0.2
HIV antibody, %	0 (0.0)	2 (9.1)	9 (4.5)	0.3
Drug in urine (March 2023)				
Benzodiazepine, %	7 (46.7)	9 (40.9)	32 (15.8)	0.002 (12.7)
Cannabis, %	5 (33.3)	5 (22.7)	16 (7.9)	0.007 (9.9)
Opioids, %	3 (20.0)	5 (22.7)	24 (11.9)	0.3
Cocaine, %	6 (40.0)	8 (36.4)	36 (17.8)	0.03 (6.8)
Pregabalin, %	5 (33.3)	7 (31.8)	15 (7.4)	<0.001 (19.1)
Methylphenidate, %	2 (13.3)	6 (27.3)	5 (2.5)	<0.001 (17.0)
Methadone (2023)				
*n*	*n* = 14	*n* = 22	*n* = 192	
Dose, mg/day	134.6 ± 23.2	132.4 ± 34.8	112.2 ± 39.0 *	0.003 (7.3, df = 2)
Serum, ng/mL	637.5 ± 191.7	765.0 ± 432.3	588.1 ± 304.5	0.17

Categorical variables are compared using the chi-square test (likelihood ratio). Continuous variables are compared using ANOVA; mean ± S.D. is presented. * Games–Howell post hoc comparisons: methadone doses were lower in the Never group than in the Always (adjusted *p* = 0.01) and Stopped/Started (adjusted *p* = 0.05) groups.

#### 3.3.2. Methadone Dose and Serum Level (Table 2)

Methadone doses in March 2023 and March 2025 differed across the three fentanyl groups, with a significant time effect (*p* < 0.001) and a significant time-by-group interaction (*p* = 0.01). Specifically, methadone doses increased among patients who consistently tested positive for fentanyl or changed fentanyl status but remained stable among patients who were consistently fentanyl-negative. A significant group effect was also observed (*p* = 0.015), reflecting lower methadone doses in the consistently fentanyl-negative group compared with the other two groups (Table 2). Evaluation of changes in serum methadone levels, adjusted for the methadone dose at the time of measurement among 170 patients, revealed no differences in serum methadone levels between groups or over time, while methadone dose showed a significant group effect with no significant time effect or time-by-group interaction.

**Table 2:** Methadone dose and serum level changes by patient groups according to their fentanyl urine-screening results in March 2023 and March 2025 (repeated-measures analyses).

	*N*	March 2023	March 2025	*p*(F) Time	*p*(F) T*G	*p*(F) Group
Methadone dose, mg/day				<0.001 (24.3, df = 1)	0.01 (4.7, df = 2)	0.015 (4.3, df = 2)
Always	15	121.0 ± 19.6	135.7 ± 26.6			
Stopped/Started	22	122.7 ± 29.8	134.3 ± 34.9			
Never	198 #	108.2 ± 37.5	111.0 ± 39.8			
		2023	2024 or 2025			
Methadone dose, * mg/day				0.3	0.07	0.02 (3.9, df = 2)
Always	10	133.0 ± 23.7	133.0 ± 23.7			
Stopped/Started	14	138.6 ± 39.4	143.9 ± 34.7			
Never	141	114.5 ± 40.0	113.5 ± 40.9			
Methadone level, * ng/mL				0.8	0.3	0.08
Always	10	673.0 ± 185.0	678.0 ± 180.1			
Stopped/Started	14	750.7 ± 476.2	787.5 ± 596.9			
Never	141	600.4 ± 306.8	576.2 ± 264.4			

# Four patients were excluded, as they were treated with buprenorphine; * methadone dose when serum level was done in 2023 and again in 2024 or 2025 (*n* = 170).

## 4. Discussion

The observed reduction in the proportion of fentanyl-positive urine tests among patients receiving MMT parallels the reduction in fentanyl availability following HMO restrictions, which were implemented in response to earlier prescribing policies that allowed a broader scope of fentanyl prescribing. Importantly, fentanyl prevalence among patients receiving MMT declined, most likely reflecting reduced availability on the illicit or diverted market. Among the cohort of patients tested in both 2023 and 2025, those who tested positive for fentanyl at both time points, referred to as “always”, had the highest proportion of concomitant substance use, with benzodiazepine 46.7%, cannabis 33.3%, cocaine 40%, pregabalin 33.3%, and methylphenidate 13.3%, compared with patients who consistently tested negative, referred to as “never”, with benzodiazepine 15.8%, cannabis 7.9%, cocaine 17.8%, pregabalin 7.4%, and methylphenidate 2.5%. Patients who discontinued or initiated fentanyl use fell between these two groups with respect to treatment duration and substance use. These findings may reflect the rehabilitation process, which requires time within treatment; however, these descriptive data need to be confirmed in a larger sample that properly addresses the question of whether dose increases precede and predict cessation of fentanyl use.

The adequate methadone dose required to achieve opioid abstinence may differ by opioid type and is generally higher among fentanyl users than among users of other opioids. Fentanyl is a highly potent opioid, with a potency of more than 50 times greater than that of heroin and morphine, resulting in the development of higher opioid tolerance compared with heroin or prescription opioids [20]. Consequently, the need for higher initial methadone doses and more rapid dose escalation has been emphasized in recent guidance from leading substance-use authorities in both Canada and the United States [21,22]. In a study examining self-reported methadone dose adequacy, individuals exposed to fentanyl were more likely to perceive their methadone dose as insufficient, while those who were exposed to non-fentanyl opioids did not report this perception, despite similar methadone doses between the groups [23].

In the current descriptive study cohort, we observed significantly higher methadone doses and a non-significant trend toward higher serum methadone levels among all fentanyl users (134.6 ± 23.2 mg/day and 637.5 ± 191.7 ng/ml, respectively), including those who screened positive for fentanyl only once, referred to as “Stopped/Started”, compared with patients who never screened positive (112.2 ± 39.0 mg/day and 588.1 ± 304.5 ng/ml, respectively). Patients who never tested positive for fentanyl (*n* = 202) were clinically stable and opioid abstinent on an adequate, relatively low methadone dose. In contrast, the small group of patients with either ongoing (*n* = 15) or intermittent fentanyl use (*n* = 22), many of whom were polysubstance users, likely required higher methadone doses. In such small numbers and follow-up, and differing with respect to concomitant substance use groups, it is impossible to learn whether some achieved adequate dosing, as reflected by fentanyl discontinuation, while others continued fentanyl misuse, suggesting ongoing underdosing. Between 2023 and 2025, the methadone doses increased among the “Always” and “Stopped/Started” fentanyl groups, while no dose change was observed among the consistently fentanyl-negative group, which may support the role of dose optimization in facilitating fentanyl cessation. Serum methadone levels were available only for a subset of patients, which limited statistical power to detect temporal changes.

While our findings regarding opioids, specifically, the significant reduction following the implementation of regulated procedures, support the importance of clear regulatory frameworks for medications with abuse potential, the absence of restrictions for other drug classes is reflected in our findings concerning pregabalin. Pregabalin (Lyrica), one of two gabapentinoid medications, is indicated in Israel for the treatment of peripheral and central neuropathic pain in adults, fibromyalgia, and generalized anxiety disorder in adults. Despite these approved indications, pregabalin is frequently prescribed for nonspecific pain complaints, often without adequate evaluation of the underlying pain etiology, adherence to the minimum effective dose, or consideration of its abuse potential and safety risks. Furthermore, prescribing information for opioid medications explicitly highlights the risks associated with concomitant use of gabapentinoids; similar warnings may be less prominent in the prescribing information for pregabalin. This issue is particularly concerning among patients receiving MMT, a population with well-documented vulnerability to substance misuse, among whom the combination of opioids, particularly methadone, and gabapentinoids has been associated with an increased risk of fatal outcomes [24,25,26]. Consistent with the lack of comparable regulatory oversight, pregabalin positivity in urine samples remained unchanged throughout the study period.

While pregabalin misuse in the general population may be driven by other factors (e.g., its legitimate prescribing for pain and its unique reinforcing effects), different policy measures may therefore be needed at the national level. Within MMT, where pregabalin use is rarely permitted under prescription, we hypothesize that its misuse may be sensitive to supply-side restrictions. Nevertheless, distinct determinants of pregabalin misuse may also operate in this population.

Collectively, these findings highlight the urgent need for clear national policies established by the Ministry of Health regarding the prescription of medications with documented addictive potential and risk of fatal outcomes, including mandatory consideration of drug–drug interactions in patients receiving polypharmacy. However, the implementation of these policies by HMOs in Israel remains incomplete, partly because of a shortage of pain specialists, and each HMO faces challenges in implementing the required procedures. In parallel, there is a need for innovative pain-management strategies, including the development of new pharmacological classes and novel therapeutic combinations of existing medications, with potential applications in pain management, to reduce reliance on high-risk medications [27,28].

### Limitations and Strengths

The strength of this study lies in the use of objective data derived from random urine drug screening, which provided an objective assessment of temporal trends in fentanyl and pregabalin positivity. However, several limitations should be acknowledged. First, the findings are derived from a single MMT clinic, which limits their generalizability. Nevertheless, this clinic is currently the only one in Israel that conducts periodic prevalence monitoring of fentanyl and pregabalin. Future studies should include larger samples, longer follow-up periods, and data from multiple MMT clinics and treatment facilities to validate and extend these findings. In addition, serum methadone level measurements were not available for all participants at both assessment time points, which may have limited the precision of analyses of methadone doses and serum concentrations.

Potential selection bias should also be considered in the longitudinal analyses. The subgroup analysis included patients who remained in MMT between March 2023 and March 2025 and had available evaluations at both time points. These patients may differ from those who discontinued treatment or lacked complete assessments, potentially limiting the representativeness of this subgroup. Consequently, the findings regarding longitudinal changes in methadone doses and serum concentrations, as well as their associations with fentanyl positivity, should be interpreted cautiously. However, the temporal prevalence trends were assessed among all patients receiving MMT at each assessment time point, rather than being restricted to this longitudinal subgroup.

Another limitation is the lack of confirmatory urine testing and prescription data. However, the clinic’s treatment protocol generally discourages or restricts the prescribing of pregabalin, methylphenidate, and benzodiazepines and does not permit opioid prescribing. Furthermore, the observational study design precludes causal inferences regarding the association between prescribing restrictions and changes in fentanyl positivity. The relatively small sample of patients with serum methadone measurements at both assessment time points also limited statistical power, particularly for multiple comparisons. In addition, the study did not include clinical outcomes.

## 5. Conclusions

The reduction in fentanyl misuse prevalence among patients receiving MMT appears to reflect decreased fentanyl availability, potentially associated with stricter prescription regulations implemented by HMOs. In contrast, the prevalence of pregabalin misuse among patients receiving MMT remained consistently high. Further research is needed to identify the distinct drivers of pregabalin misuse and evaluate appropriate prevention and prescribing strategies. Patients with fentanyl-positive urine screens may require higher methadone doses.

## Data Availability

The data presented in this study are available on request from the corresponding author due to privacy.

## Abbreviations

The following abbreviations are used in this manuscript:

HMOs	Health Maintenance Organizations
MMT	Methadone Maintenance Treatment
OUD	Opioid Use Disorder
THD	Take-Home Methadone Dose
DSM	Diagnostic and Statistical Manual
GEE	Generalized Estimating Equations
ANOVA	Analysis of Variance
